# Data-Driven Modeling and Design of Sustainable High Tg Polymers

**DOI:** 10.3390/ijms26062743

**Published:** 2025-03-18

**Authors:** Qinrui Liu, Michael F. Forrester, Dhananjay Dileep, Aadhi Subbiah, Vivek Garg, Demetrius Finley, Eric W. Cochran, George A. Kraus, Scott R. Broderick

**Affiliations:** 1 Department of Materials Design and Innovation, University at Buffalo, Buffalo, NY 14260, USA; 2 Department of Chemical and Biological Engineering, Iowa State University, Ames, IA 50011, USAdjay11@iastate.edu (D.D.); asubbiah@iastate.edu (A.S.); dafinley@iastate.edu (D.F.);

**Keywords:** sustainable polymers, glass transition temperature, topological descriptors, machine learning, graph theory

## Abstract

This paper develops a machine learning methodology for the rapid and robust prediction of the glass transition temperature (Tg) for polymers for the targeted application of sustainable high-temperature polymers. The machine learning framework combines multiple techniques to develop a feature set encompassing all relative aspects of polymer chemistry, to extract and explain correlations between features and Tg, and to develop and apply a high-throughput predictive model. In this work, we identify aspects of the chemistry that most impact Tg, including a parameter related to rotational degrees of freedom and a backbone index based on a steric hindrance parameter. Building on this scientific understanding, models are developed on different types of data to ensure robustness, and experimental validation is obtained through the testing of new polymer chemistry with remarkable Tg. The ability of our model to predict Tg shows that the relevant information is contained within the topological descriptors, while the requirement of non-linear manifold transformation of the data also shows that the relationships are complex and cannot be captured through traditional regression approaches. Building on the scientific understanding obtained from the correlation analyses, coupled with the model performance, it is shown that the rigidity and interaction dynamics of the polymer structure are key to tuning for achieving targeted performance. This work has implications for future rapid optimization of chemistries

## 1. Introduction

In recent decades, petroleum-based plastics, sourced from fossil fuels, have dominated the production of commodity plastics; however, only about 9% of plastic waste has been recycled. The increasing ecological and public health concerns have emphasized the need for developing sustainable polymers [1]. Although the definition of sustainable polymers remains somewhat open to interpretation, it generally encompasses polymers derived from biomass-based monomers, those synthesized using green chemistry principles, polymers capable of closed-loop chemical recycling (including monomer recovery, repurposing to alternative materials, or degradation via biological or simulated environmental processes), and those for which a comprehensive life cycle assessment has been performed [2,3,4].

Bio-renewable polymers, a subset of sustainable polymers, are materials in which at least part of the polymer is produced from renewable raw materials, such as biomass from sugarcane or corn [5]. These polymers can be derived from biochemical processes involving plant sources (e.g., cellulose-based polymers, alginate, polyisoprene, starch) and animal sources (e.g., polylactic acid, polyhydroxyalkanoates, polybutylene succinate), as well as bacterial fermentation products (e.g., chitin, chitosan, collagen, sericin) [6,7]. Modifying bio-renewable polymers by incorporating different functional groups can yield a variety of derivatives [8,9]. It is important to note that not all bio-renewable polymers are biodegradable, as a polymer’s biodegradability is determined by its chemical structure rather than the source of its raw materials [2,3]. The conversion of biomass-based feedstocks into polymers not only reduces reliance on fossil fuels but also offers numerous opportunities to design bio-renewable polymers with tailored properties and functionalities.

In real-world applications, biopolymers face certain limitations due to their relatively low mechanical strength and thermal stability. The glass transition temperature (Tg) is a critical indicator of a polymer’s thermal properties, marking the point where a polymer transitions from a rigid, glassy state to a softer, rubbery state. Polymers with higher Tg values exhibit enhanced thermal stability, allowing them to maintain their mechanical integrity even under elevated temperatures. This makes them ideal for use in high-performance applications such as energy, aerospace, biomedical, and electronics [10,11], where materials might withstand extreme thermal environments.

The majority of commonly used biopolymers have Tg values ranging from 55 °C to 75 °C [2,12,13]. Focusing on the discovery of high Tg polymers is essential to meet the growing demands for materials that can withstand challenging operational conditions without compromising functionality, as well as remaining environmentally friendly. Numerous studies have investigated the structural design of backbones and side groups in the pursuit of polymers with higher Tg. Most of these efforts have focused on synthesizing novel polymers through the manipulation of monomer ratios and crosslinks, introducing additional functional groups [14,15,16,17], as well as exploring various polymerization methods to synthesize polymers with tunable Tg values [18]. These prior studies suggest that high Tg polymers often have rigid backbones that limit chain flexibility. A common strategy to increase rigidity involves adding non-flexible ring structures to the backbone, as seen in polymers like aromatic polycarbonates, polynorbornenes, and polyimides. Another approach is introducing substituents near the backbone to hinder chain rotation via steric interactions, exemplified by poly (α-methyl styrene) and similar polymers [19,20,21].

While these investigations have provided valuable insights for further development, they are often constrained by experimental capacity and may not fully capture the complexity of factors that influence polymer properties. Additionally, many of these case-by-case studies include specific types of chemical groups, as the analyses typically rely on comparisons between polymers with similar main-chain structures [12,22]. To overcome these limitations, quantitative and systematic approaches are needed to evaluate the structure–property relationships of polymer candidates. Our goal is to utilize an unbiased approach that examines all topological features derived from monomer chemical structures, in order to systematically identify the key factors governing Tg.

There have been multiple methods developed to establish an efficient descriptor base, including Bicerano’s topological indices [23], Van Krevelen’s additive group contributions [24], and Askadskii’s semi-empirical method based on Van der Waals volume and dispersion interaction [25]. Other relevant recent approaches include the polymer genome database [26,27], polymer quantum chemistry combined with machine learning [28], predictions based on SMILES string of polymers [29], empirical QSAR data for polymers [30,31], and hybrid descriptors [32]. With the advancement of machine learning (ML), an increasing number of studies have been conducted in the material design field integrating these polymer structure descriptors to accelerate the prediction of polymer properties [33,34,35,36,37,38,39,40,41,42,43]. In this paper, we build upon these works, as well as our additional works in ML [44,45,46,47,48], to develop a model capturing the underlying physics to accelerate the discovery of high Tg renewable polymers.

## 2. Results

### 2.1. Data

In order to develop a descriptor set that encompasses multiple aspects of the structure in a bias-free manner, the components of the molecular structure were calculated (Table 1). The logic of this approach is that the descriptors should be physically meaningful, while being calculable for any structures, thereby allowing for improved mechanistic understanding and robust modeling of new polymer systems. The descriptor set was built based partially on the descriptors defined by Bicerano [23] and Van Krevelen [24]. On the basis of graph theory, Bicerano defined topological connectivity indices and used them to predict more than 70 polymer properties. Building on this logic, with the inclusion of ML analyses, it is expected that the information gain and property prediction can be further expanded and enhanced. The process for descriptor calculation, as well as a more thorough description of the features, is provided in the Supplementary Materials.

The values for Tg, which are used in the analysis, were taken from two different sources. The first was from assorted literature values, as compiled in the “Polymer Products from Aldrich” catalog [49]. These data were for commonly available polymers, with the data spanning multiple sources. From these data, we calculated the topological features of approximately 80 polymer chemistries. When multiple Tg values (or a range of values) were provided for a given polymer, the average value was taken. This approximation, along with the combination of various sources, introduces uncertainty and error into the analysis; however, the range of approaches used and the consideration of underlying science provide sufficient confidence for this approach. The second set of Tg values was focused on biobased acrylates [50]. This data set provides a smaller data set; however, by being centered on the acrylate systems, it provides a more focused examination in terms of the relevance to sustainable polymers. The first data set was used in the correlation analyses, while both data sets were independently used in the predictive modeling. The reason for using two data sets is to ensure that the model is robust and not specific to a certain data type or range of systems, or requiring a specific number of measurements. Additionally, comparing the two models allows for assessing if there are differences in applicability between acrylate-focused data and broader data. In this regard, if the broader data provide similar accuracy as unseen acrylate data, then the broader data can be used in the future, as the larger data size will help reduce uncertainty.

One consideration to raise is that polymers with the same chemical composition can have different microstructures and very different Tg values. To account for this, our model incorporates descriptors that capture structural and chemical influences on Tg, including features related to molecular flexibility, intermolecular interactions, and steric effects. While microstructural variations are not explicitly parameterized, their impact is indirectly reflected in the selected features, such as rotational freedom, hydrogen-bonding potential, and aromaticity, all of which influence polymer packing and rigidity. A more explicit characterization of microstructure (e.g., crystallinity levels) could further refine the predictive power of the model and reduce uncertainty.

### 2.2. Data-Driven Modeling

**Figure 1:**
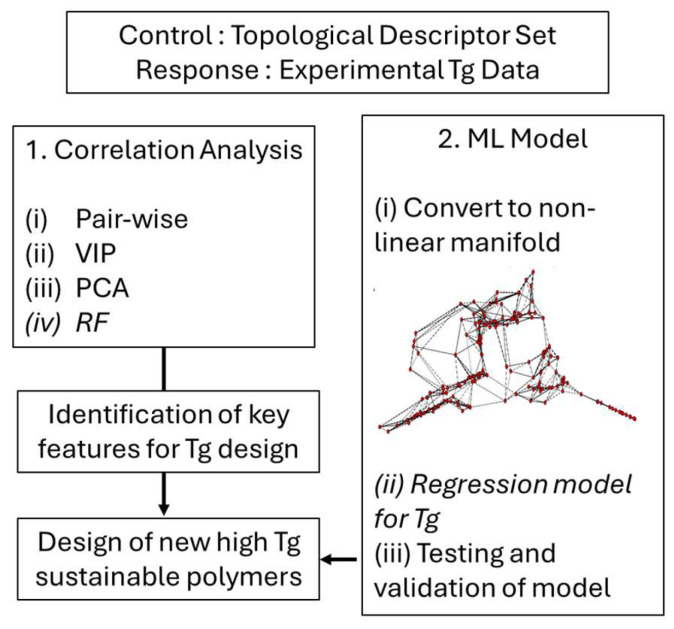
Logic of this paper. The input feature set is based on the topological descriptors, with the work having two aspects: correlation analysis for scientific reasoning and prediction through ML modeling.

Two different aspects of modeling were applied: unsupervised learning to understand the underlying correlations and science in the data, and supervised learning to develop predictive models for Tg. The consideration of both aspects is significant because it allows for both a high-throughput model but also provides a science-driven description. An additional novelty of this work is the integration of unsupervised learning and supervised learning. In the case of the correlation analyses, the logic followed an unsupervised approach, but some supervised learning was integrated into the analysis. For the supervised learning approach (predicting Tg), the feature set was first converted into a non-linear parameterization using an unsupervised methodology. Figure 1 summarizes the logic, with the first part (Section 2.3) focused on the correlation and understanding of the features described above with Tg, and the second part (Section 2.4) focused on the development and testing of a quantitative model. The future application of optimized polymer design is discussed.

### 2.3. Correlations

**Figure 2:**
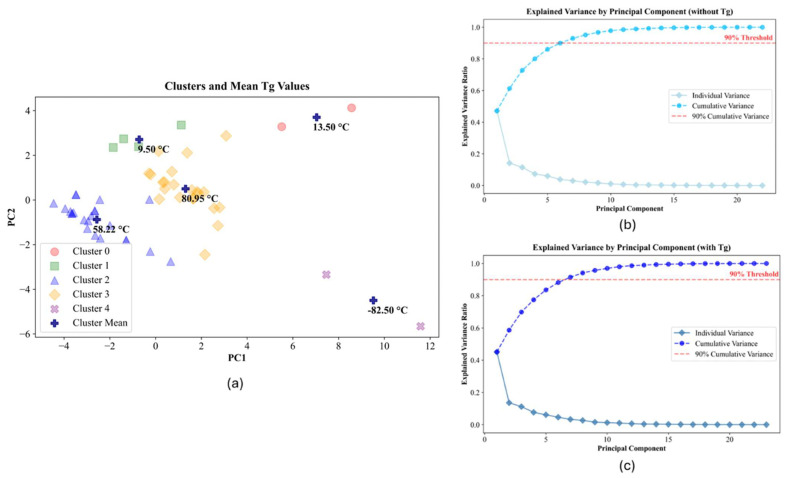
PC analysis to ensure feature set (Table 1) captures changes in Tg. (**a**) Average values of Tg for each cluster defined in the PCs through k-means clustering are shown. These demonstrate that PCA captures the different chemistries resulting in different Tg values. (**b**) Variance of each PC without Tg included in the analysis. (**c**) Variance of each PC with Tg included in the analysis. The similarity of (**b**,**c**) shows that Tg is sufficiently captured in the feature set.

The comparison between Tg and the features (Table 1) was assessed through a variety of methods to ensure robustness in conclusions and to verify that no conclusion or interpretation is based solely on the methodology applied. As a first step, we want to ensure that Tg is captured by the feature set. If not, then any model would be expected to have little applicability. To test whether Tg is in fact somehow captured in the correlations within the data, PCA was applied with only the features (and not Tg values) and then repeated with Tg included. The purpose of this step is to see if the variability captured in PCA changes significantly with the addition of Tg; in that case, the result would be based primarily on statistical fitting, suggesting Tg is not represented well in the features and additional features are needed. Figure 2 shows the results of this analysis. The first step (Figure 2a) was to apply k-means clustering to the Principal Component (PC) results. The k-means step identifies those chemistries that are most similar, as defined by proximity in PC space. For this analysis, Tg was not input as the purpose was to explore if Tg is captured without being defined. The labels on the figure are the average Tg for each cluster. Significantly different values in Tg are captured in each cluster, suggesting that Tg is in fact represented in the features and captured in the analysis. The standard deviation in Tg for each cluster is also relatively consistent, so the values are not based on outliers or a few large values. The comparison of variance captured by the PCs, both without and with Tg included (Figure 2b,c, respectively), shows the variance is consistent; thus, the inclusion of Tg does not provide significantly new information. These results indicate that the feature set developed is sufficient for assessing and modeling Tg.

**Figure 3:**
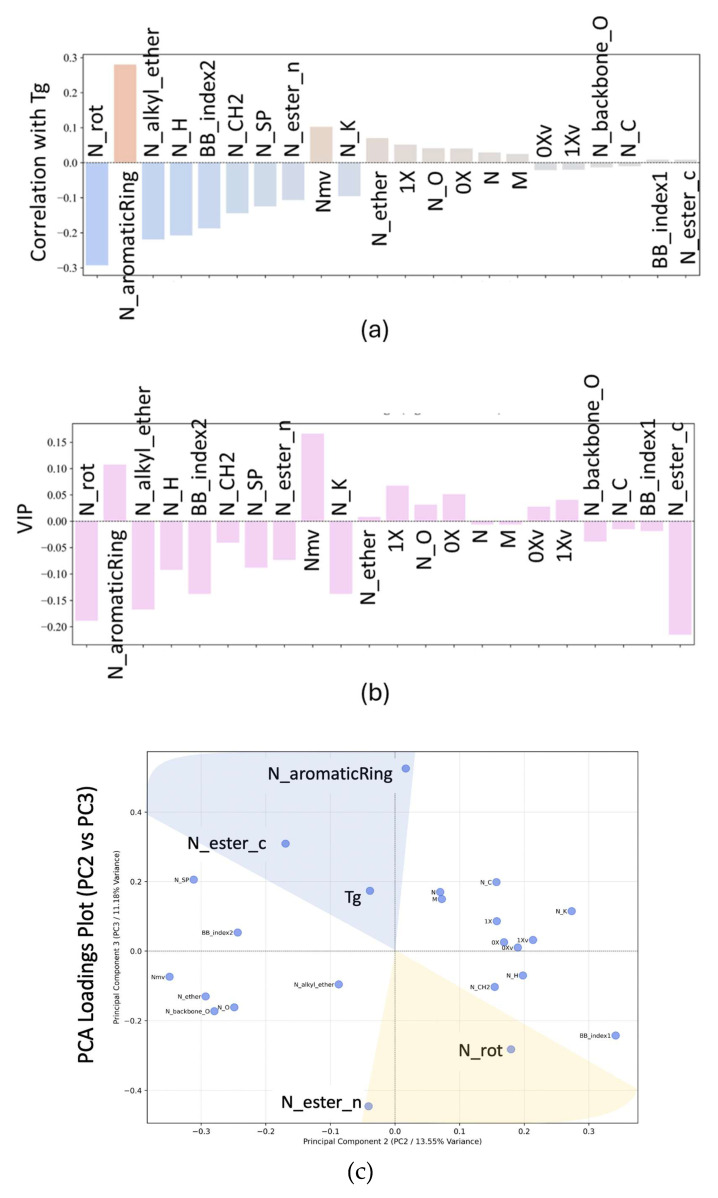

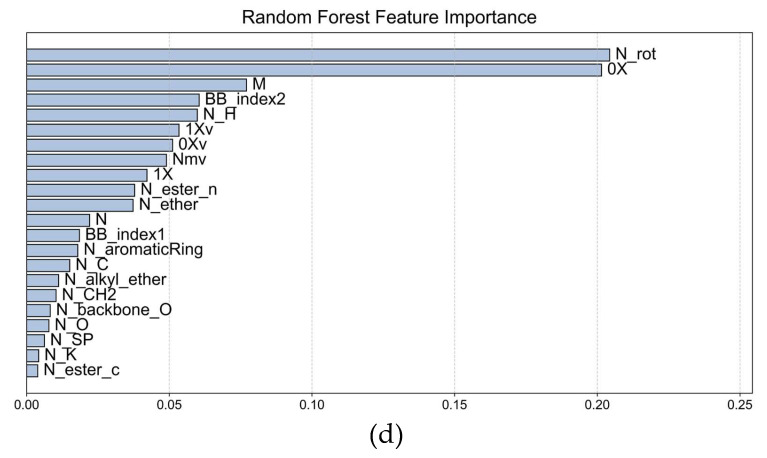
Results from correlation analyses. The labels correspond with Table 1. (**a**) Pair-wise correlations, (**b**) VIP result, (**c**) PCA loading plot with Tg-correlation areas shaded, and (**d**) Random Forest feature importance.

Four different approaches for calculating correlations were applied: pair-wise correlation, VIP for five PCs, the visual comparison of points within PC space for the first three PCs, and the importance of the features from the RF model. The results of these correlations are shown in Figure 3. Positive values indicate a positive correlation, while negative values indicate an inverse correlation. It should be noted that the data set here only contained 53 data points; however, the issue is not necessarily in the size of the data, but rather that the broad range of relevant information is contained in the data. Further to ensure that the results were not over-fit to a relatively small data, the correlation results were tested for robustness by removing data and ensuring that the conclusions did not change. Additionally, in the next section, the development of a reasonable predictive model using these data gives confidence that the relevant underlying relationships are represented in these data.

The results across the methods are fairly consistent, with the only significant difference being that VIP identifies N_ester_c as having a large inverse correlation. The inclusion of noise in the lower PCs does not significantly impact the conclusions when comparing Figure 3a,b. The cumulative results of all four approaches are shown in Table 2. In this table, the number of times that a feature was identified among the four approaches is listed, as well as if the correlation was positive (+) or inverse (−).

Since each method highlights different aspects of feature importance, we combined the results and counted how frequently each feature was selected across the four techniques. The “Times Selected” column in Table 2 reflects the number of methods that identified a given feature as important. For the pair-wise correlation, VIP, and RF analyses, the top six features with the strongest relationships to Tg were selected, while the PCA loadings features were identified based on their positioning within a predefined shaded cone, capturing those with significant correlation with Tg. While the cut-offs could be defined differently, based on the visual analysis of the resulting correlations, it was determined that this cut-off was sufficient to capture the key correlations without including weak correlations.

In terms of conclusions, N_rot was identified as important, with a negative correlation, by all four approaches. BB_index2 was identified as inversely correlated by three of the four models. This would indicate that these two features are most important in controlling Tg. In the case of N_ether_c, it was identified by different models as having different impacts, while the last three features were identified by the RF model, which does not give directionality of correlation. In the Supplementary Materials, heat maps showing the correlations between features are included. From these heat maps, it is found that there are some strong intercorrelations in these key features. N_H, ^1^X^v^, and ^0^X^V^ are all highly correlated, and thus the information added by including more than one is not that significant. It is noteworthy that many of the topological descriptors are driven by the number of hydrogen atoms. Nmv and N_K also have strong inverse correlations.

Following these results, several interpretations are made. Achieving a high Tg in polymers involves multiple interconnected strategies that target the rigidity/flexibility and interaction dynamics of the polymer structure. One effective approach is the incorporation of conformationally rigid components, such as aliphatic or aromatic rings, into the polymer backbone. These rigid elements reduce chain flexibility, directly contributing to an increase in Tg [8,51]. Additionally, the choice of side-chain structures plays a critical role; side groups designed to lower chain mobility can stabilize the polymer, further enhancing Tg [2].

Another important factor is the introduction of high conformational barriers along the polymer backbone. Structural modifications, such as additional substituents or sp^2^-hybridized carbons, create these barriers, reducing the effective degrees of freedom and elevating Tg [20]. Furthermore, augmenting chain–chain interactions through the use of polar or hydrogen-bonding groups strengthens inter-chain forces, which also contribute to a higher Tg. These interactions are better observed in the connected polymerized structure than in individual monomers [52]. Aromatic segments are particularly advantageous in achieving high Tg due to their intrinsic rigidity and capacity for strong inter-chain interactions. Incorporating “hard” segments with high conformational barriers and robust polar associations, such as π–π stacking, effectively enhances Tg. Compared to aliphatic segments, aromatic components impose stricter conformational constraints and facilitate stronger polar interactions, making them highly effective in boosting thermal stability [2,8,52].

The description of these key concepts aligns with the explanation of free rotational degrees commonly discussed in the literature and research. Increasing the rigidity of components, reducing chain mobility, and raising conformational barriers directly affect the number of bonds that can be classified as ’free’ single bonds in terms of rotational degrees. The incorporation of rigid rings, floppy rings, or semi-floppy rings into the polymer backbone or side groups modifies the structural dynamics, as these elements do not contribute rotational freedom in the same manner as single bonds [23]. Notably, these structural changes have a minimal impact on the number of atoms connected to the bonds, preserving overall atomic stability while enhancing rigidity.

### 2.4. Tg Prediction

Two different models were developed for predicting Tg. The first approach used a larger data set that contained a wide variety of polymer chemistries [49], while the second data set was smaller but focused on acrylate systems that are more relevant to our stated objectives [50]. A comparison of the two defines if we can develop a larger and more generalized model that is applicable to all systems and the level of confidence in using the models to design high-temperature sustainable polymer chemistries.

**Figure 4:**
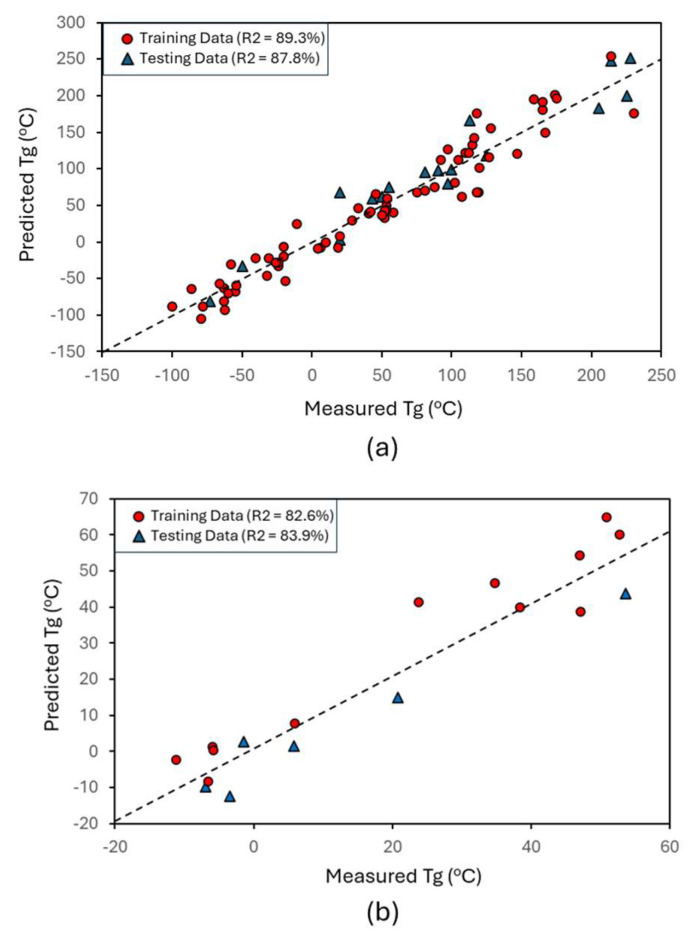
Parity plot from the models, where a regression was applied to the IsoMapping of the feature space. (**a**) Model trained to a larger, more general data set. (**b**) Model trained to a smaller data set focused on acrylates. The similarity in model accuracies demonstrates the robustness of the ML framework. Note that the axes are different within and across the plots for visualization reasons.

As discussed, the feature set was first converted into a non-linear manifold and then a multi-linear regression was applied. The results of the larger data are shown in Figure 4a. The model has high accuracy, especially considering the uncertainty within the data. A total of 20% of the data were removed prior to building the model and were used as testing data. As seen in the figure, the accuracy of the model was comparable to the testing data, demonstrating that the model was not over-fit and is robust. The process was repeated with the smaller acrylate data set (Figure 4b). Again, the accuracy was high, with comparable training and test accuracy, as shown in the R^2^ values labeled in Figure 4. In both cases, the accuracy between training and test data is similar, showing that the models are not overfitting the data and are driven by the underlying relationships in the data. The lower accuracy is expected, given the smaller data set. However, the application of the same feature set and ML framework were applied in both cases, with similar results, demonstrating that the feature set sufficiently captures the mechanics of Tg for polymers in general and acrylates specifically. Based on this, it is anticipated that the larger (or combined) data sets can be used in the future, even when focusing on specific polymer types. This agrees with the result of Figure 3a, where it was shown that different regimes of Tg map together. Although in that case, it was PC space and not the non-linear space used in the prediction, the concept of self-organization in systems is still relevant.

## 3. Discussion

**Figure 5:**
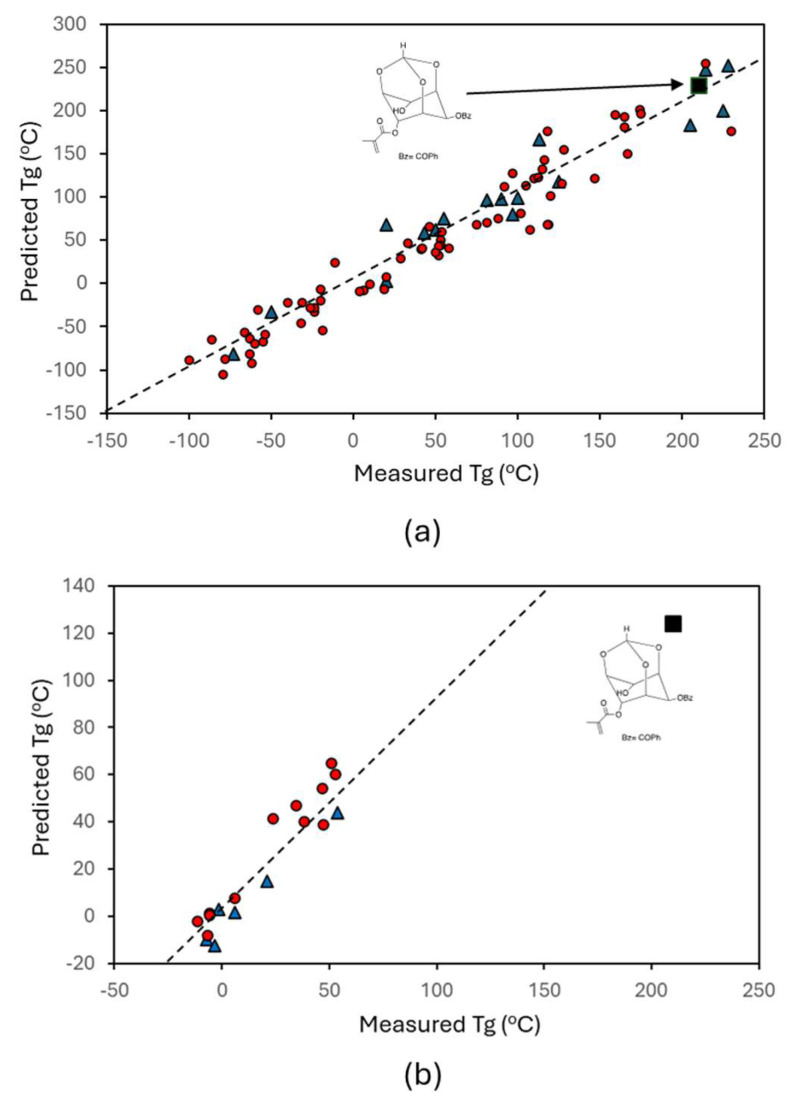
Predictive models corresponding with Figure 3, but with an additional new experimental point added, with the monomer chemistry shown in the figures and the chemistry shown as a square. The model with the larger data (**a**) predicts this chemistry well, while the smaller acrylate data (**b**) predicts the behavior well qualitatively.

To experimentally validate our model, it was applied to a recently experimentally processed and measured chemistry (Figure 5). The material is available from myo-inositol, a natural material. The polymer was measured to have a remarkable Tg of 210 °C. Relevant experimental details of the process were discussed in our prior work [50]. This chemistry was subsequently predicted using both models. In the case of the larger data model, the Tg value was predicted with high accuracy. In the smaller acrylate data set, the predicted value of the new experimental polymer is not particularly accurate. However, most predictions are not good with extrapolation, as compared with interpolation. That is, models work much better where we have data and are less accurate where we do not have data (such as in this case, where the Tg value is much higher than anything included in the model). However, the model still qualitatively works quite well, as it predicts a much higher Tg for it than any of the other systems. While quantitatively the prediction is not highly accurate, qualitatively it is still accurate and performs within the expected uncertainty in that region, given the limited data included at those Tg ranges. Both results provide a point of experimental validation.

## 4. Materials and Methods

### 4.1. Unsupervised Learning

To develop correlations between the features and Tg, multiple approaches were used, with principal component analysis (PCA) being the main tool considered. PCA converts the data into linear combinations of the features, with the combinations based on capturing the largest amount of independent variance in the data. In this way, the correlations and information in the data are captured in fewer dimensions. Since the feature set developed in this work is largely correlated, using it as is would result in a model that has large correlations; therefore, the model would be based solely on statistical relationships and not capture the underlying science. To assess the correlations, a pair-wise correlation between each feature and Tg is made. This is followed by calculating the dot product between Tg and each feature after being converted into PC space, providing a so-called Variable Importance Projection (VIP). The VIP serves as akin to a pair-wise correlation except in only a limited number of PCs. Finally, correlations are made based on a supervised learning approach, described in that section.

An unsupervised methodology is also integrated into the predictive modeling. Through the use of IsoMap, the data are converted onto a non-linear manifold. This approach is akin to PCA; however, by considering the geodesic distances, non-linear relationships can be captured. The primary reason for including this step is that it was found to significantly improve model accuracy. This indicates that the relationships between features and Tg are better captured in non-linear space. Additionally, given the model accuracy, we can conclude that the relationships described through the various correlative analyses are still maintained in this approach. The negative aspect of this step though is that we lose interpretability as to what the model is capturing. However, since we are including the previous step of assessing correlations in the data through different correlative analyses, we still have a scientific assessment of the models.

### 4.2. Supervised Learning

The final models developed were built solely through the use of multiple linear regression. The key step in the model was the conversion of the features first into the non-linear parameterization with IsoMap. The logic in selecting multiple linear regression is that the simplest model that provides high-quality results is the most likely to maintain robustness. Therefore, this approach is efficient, accurate, and applicable to a broad range of chemistries and structures. Additionally, random forest (RF) analysis was applied between the features and Tg. The model performed relatively poorly, as compared to the multiple linear approach applied to IsoMap parameterization. However, it is still suitable for an additional measure of correlations, where the features that most impact the RF model are identified and compared with the correlation measures through the unsupervised aspect.

## 5. Conclusions

In this paper, a variety of unique aspects were combined into a single machine learning framework for the prediction and design of sustainable high-Tg polymers. This framework included the development of a feature space based on topological descriptors that can be calculated for any chemistry. The key features controlling Tg were identified and reasonably understood, while it was also verified that the features contained relative information on Tg, thereby leading to an analysis based on underlying science and not statistical fitting. The assessment of features was based on an integration of unsupervised and supervised approaches, while a separate aspect focused on the quantitative prediction of Tg. Using two different data sets, the Tg was well predicted and was additionally experimentally validated with a new, high-Tg sustainable polymer chemistry. This work has significant future implications, first for the automation of the process, and secondly for the optimization of polymers for target properties. While this paper has focused on Tg, it can be expanded in the future to consider other properties and the underlying trade-offs. Therefore, this paper has introduced a machine learning approach for assessing and predicting high-temperature polymers, based on both scientific basis and predictions, while laying out future applications for accelerated design and optimization of multi-functional polymer chemistries.

## Figures and Tables

**Table 1:** The feature set developed and calculated for the relevant polymers. These features are then used in the ML framework for Tg modeling.

Polymer Features	How They Were Defined or Calculated
N	total non-hydrogen atoms in one polymer repeat unit
N_C	number of carbon atoms in one polymeric repeat unit
N_H	number of hydrogen atoms in one polymeric repeat unit
N_ester_n	number of backbone -COO- (non-conjugated with aromatic ring)
N_ester_c	number of backbone -COO- (one-sided conjugation with aromatic ring)
N_aromaticring	number of aromatic rings in one polymeric repeat unit
N_CH2	number of -CH_2_ in one polymeric repeat unit
N_ether	number of -O- in a polymeric repeat unit
N_backbone_O	number of backbone oxygen atoms in one polymeric repeat unit
N_O	number of oxygen atoms in a polymeric repeat unit
M	mole weight of one polymer repeat unit(g/mol)
N_alkyl_ether	number of ether (R-O-R’) linkages between two units R and R’ both of which are connected to the alkyl carbon atom
N_rot	total number of rotational degrees of freedom parameter (N_rot = the backbone rotational degrees plus the side group rotational degrees)
N_K	N_K = 5N_amide + 7N_cyanide + 15N_carbonate + 5N_Cl + 13N_Br + 4N_hydroxyl − 3N_(ether) − 5N_C = C + 3N_sulfone − 3N_acrylic ester − 5N_ (isolated saturated aliphatic hydrocarbon rings, i.e., cyclohexyl or cyclopentyl)
N_SP	number of atoms in the shortest path across the backbone of a polymeric repeat unit, N_SP ≤ N_BB
Nmv	Nmv = 2 × N_ester + 3 × N_ether
^0^Χ	the zeroth-order (atomic) connectivity indices (the first atomic index)
^0^Χ^V^	the zeroth-order (atomic) connectivity indices (the second atomic index)
^1^Χ	the first-order (bond) connectivity indices (the first bond index)
^1^Χ^V^	the first-order (bond) connectivity indices (the second bond index)
BB_index1	backbone index1 is a steric hindrance parameter that reflects the flexibility of the polymer backbone structure, similar to the stiffness of the backbone.
BB_index2	backbone index2 is a steric hindrance parameter that differentiates between backbone atoms with the same (δ/δV) values but different δ values, reflecting variations in the number of non-hydrogen neighbors around each backbone atom.

**Table 2:** Combined results for the four different methods of correlation. ‘Feature’ corresponds with the labels in Table 1, ‘Times Selected’ corresponds with the number of methods that identified the feature as important, and ‘Correlation’ labels whether the correlation was positive or inverse.

Feature	Times Selected	Correlation
N_rot	4	−
BB_index2	3	−
N_alkyl_ether	2	−
N_ether_c	2	+ and −
N_aromaticRing	2	+
N_H	2	−
Nmv	1	+
N_K	1	−
N_ester_n	1	−
^0^X	1	
^1^X^v^	1	
M	1	

## Data Availability

The data are available by reasonable email request to the corresponding author.

## Supplementary Materials

The following supporting information can be downloaded at: https://www.mdpi.com/article/10.3390/ijms26062743/s1.
