# Supplementary material for: Data-Driven Modeling and Design of Sustainable High Tg Polymers
Source: Int J Mol Sci. 2025 Mar 18;26(6):2743. doi: 10.3390/ijms26062743 (PMC11942765; doi:10.3390/ijms26062743)
Supplement: Supplementary file 1 [file ijms-26-02743-s001.zip › ijms-3501360-supplementary.pdf]

<u>**Supplementary Material**</u>

Data-Driven Modeling and Design of Sustainable High Tg Polymers

Qinrui Liu[1], Michael F. Forrester[2], Dhananjay Dileep[2], Aadhi Subbiah[2], Vivek Garg[2], Demetrius Finley[2], Eric W. Cochran[2], George A. Kraus[2], Scott R. Broderick[1,*]

[1]Department of Materials Design and Innovation, University at Buffalo, Buffalo, NY 14260, USA.
[2]Department of Chemical and Biological Engineering, Iowa State University, Ames, IA 50011, USA.
*Correspondence: scottbro@buffalo.edu

S1. Experimental Details

Monomer Synthesis: *o-Bz Cage:* Myo-inositol (2.7 g, 15 mmol), triethyl orthoformate (2.39 g, 22.5 mmol), pTSA (0.25 g, 1.31 mmol) and 20 mL of DMF are refluxed for 3 hours. The solution is cooled to room temperature, and 1 mL of triethylamine is added. DMF is removed by rotary evaporation. To remove water, azetropic distillation with toluene is done twice. The crude material is dissolved in 10mL of pyridine, and cooled to 0 °C. Benzoyl chloride (4.4 g, 30 mmol) is added dropwise over 15 minutes. The solution is allowed to warm to room temperature and stirred overnight. The dibenzyl product precipitates out of solution and is collected.

Methacrylated Dibenzoyl Bananin: o-Bz Cage (1.00g, 3 mmol) was dissolved in 10 ml chloroform. Triethylamine (0.635 g, 0.00628 mol, 2.5 equiv) and DMAP (0.080 g, 0.00020 mol, 0.080 equiv) were added to the solution. Methacrylic anhydride (0.503 g, 0.00326 mol, 1.3 equiv) was then added dropwise at a rate of (~1 mg/ml) while maintaining T below 25 °C. The reaction mixture was then heated to 40 °C and stirred at 300 rpm for 24 hours. The reaction mixture was cooled to room temperature and washed sequentially with 1M HCl, saturated NaHCO3 solution, and brine. The organic layer was dried over MgSO4, filtered, and concentrated under reduced pressure. The crude product was further dried under high vacuum at 60 °C for 3 hours to afford the methacrylated bananin as a white solid.

Polymer Synthesis: The methacrylated oBz-Cage (0.2 g) was dissolved in toluene (0.3 g, 1.5 weight ratio) in a dry, UV-protected amber 4-dram vial. The vial was purged with argon at for 5 minutes. A stock solution of AMBN in toluene was prepared by dissolving 1 g of AMBN in 88.765 g of toluene. AMBN solution (0.03 g) was added to the vial via syringe through a septum. The vial was then heated at 80 °C for 5 hours to ensure solubility of both the monomer and the polymer. Upon reaction completion and cooling the polymer starts precipitating. The polymer is then fully precipitated using hexanes. The purified polymer is dried in vacuo at 60 °C for 16-24h.

Polymer Analytics: *GPC:* Polymer molecular weights and their distributions were determined using a Waters Size Exclusion Chromatography (SEC) system. The setup included an isocratic solvent manager (ACQ-ISM), UV detector (ACQ-UV), refractive index detector (ACQ-RI), and five ethylene-bridged hybrid (BEH) packed columns connected in series: XT900A APC (300,000–2,000,000), XT450A APC (20,000–400,000), two XT200A APC (3,000–70,000), and XT45A

APC (200–5,000). Tetrahydrofuran (THF) was used as the eluent at 25 °C, with a flow rate of 0.8 mL/min. Calibration was performed using polymethyl methacrylate (PMMA) standards. Samples were prepared by dissolving 5 mg of polymer in 1 mL of THF and filtered through a 0.45 µm PTFE filter before injection.

DSC: Differential scanning calorimetry was performed on (DSC, TA instruments Discovery 2500) to conduct a thermal analysis of the polymer. Nitrogen was used as a purge gas at 50 ml/min. Samples were weighed and sealed in hermetically sealed aluminum pans. Each cycle consisted of heating the sample from 25°C to 300°C (ramp rate 10°C/min), isothermal equilibration for 5 minutes, followed by cooling the sample back to 25°C (ramp rate 10°C/min). Two cycles were performed for each dataset.

Results:

Sample	Solvents	Mn(kDa)	Mw (kDa)	Tg (°C)
Rad1	DMF+toluene	11.05	15.1	240-243
Rad2	toluene	38.89	72.96	240-243

Table: Results of radically produced methacrylated dibenzoyl bananin showing molecular weight and thermal characteristics.

S2. Feature Description

The features were defined in the ways below:

Polymer Features	How they were defined or calculated
**N**	total **non-hydrogen atom** number in one polymer repeat unit<br>(Count the non-hydrogen atoms by looking at the monomer structure)
**N_C**	number of **carbon atoms** in one polymeric repeat unit<br>(Count the carbon atoms by looking at the monomer structure)
**N_H**	number of **hydrogen atoms** in one polymeric repeat unit<br>(Count the hydrogen atoms by looking at the monomer structure)
**N_ester_n**	number of **backbone -COO- (non-conjugated with aromatic ring**)<br>(Count the -COO- structure which is not connected to any aromatic ring by looking at the monomer backbone structure)

	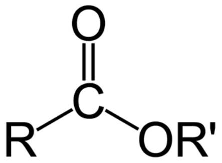
**N_ester_c**	number of **backbone -COO-** (**one-sided conjugation with aromatic ring**)<br>(Count the -COO- structure which is connected to aromatic ring by looking at the monomer backbone structure)<br>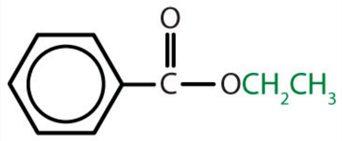
**N_aromaticring**	number of **aromatic rings** in one polymeric repeat unit<br>(Count the aromatic rings by looking at the monomer structure)<br>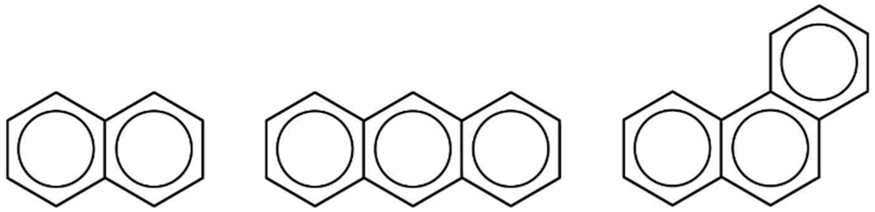
**N_CH2**	number of **-CH2** in one polymeric repeat unit<br>(Count the -CH2 structure by looking at the monomer structure)
**N_ether**	number of **-O-** in a polymeric repeat unit<br>(Count the -O- structure by looking at the monomer structure)
**N_backbone_O**	number of **backbone oxygen atoms** in one polymeric repeat unit<br>(Count the oxygen atoms by looking at the monomer backbone structure)
**N_O**	number of **oxygen atoms** in a polymeric repeat unit<br>(Count the oxygen atoms by looking at the monomer structure)
**M**	**mole weight** of one polymer repeat unit(g/mol)
**N_alkyl_ether**	number of **ether (R-O-R') linkages** between two units R and R' both of which are **connected to the alkyl carbon atom**<br>(Count the ether between 2 alkyl group carbon by looking at the monomer structure)<br>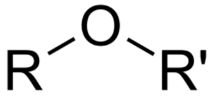

<table>
<tr><td></td><td>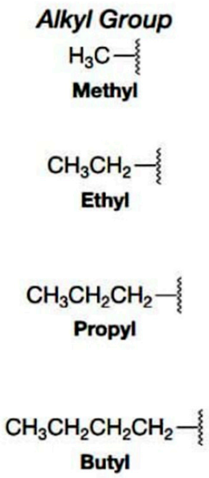
</td></tr>
<tr><td>N_rot</td><td>total number of rotational degrees of freedom parameter
N_rot = N_BBrot + N_SGrot
(N_BBrot is the backbone rotational degrees, N_SGrot is the side group rotational degrees)

Figure illustration of polymer backbone denoted in black and side group denoted in red:
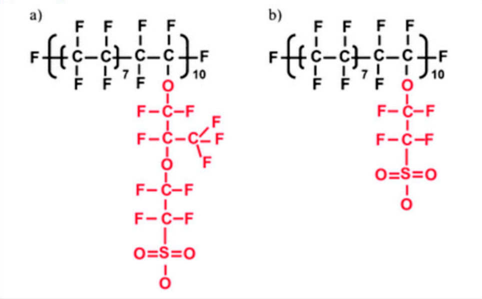

Detailed way of counting:
a. Each single bond in the backbone contributes +1 to N_BBrot, provided that this single bond is not in a ring.
b. Each single bond in a side group, or connecting a side group to the backbone, contributes +1 to N_SGrot.
c. If the coordinates of all atoms remain unchanged on rotation of a single bond in a side group, or rotation of a single bond connecting a side group to the backbone, such a single bond does not contribute to N_SGrot.
d. Multiple bonds, either in the backbone or in side groups, do not contribute either to N_BBrot or to N_SGrot.
e. Bonds with “rigid” rings, either in the backbone or in side groups, do not contribute either N_BBrot or to N_SGrot.
f. Torsional motions around bonds in “floppy” rings are generally more restricted than motions around bonds which are not in rings, but less restricted than motions around bonds in “rigid” (especially aromatic) rings:</td></tr>
</table>

	Each single bond in a “floppy” ring in the backbone contributes +0.5 to N_BBrot.<br>Each single bond in a “floppy” ring in a side group contributes +0.5 to N_SGrot.
**N_K**	**N_K = 5N_amide + 7N_cyanide + 15N_carbonate + 5N_Cl + 13N_Br + 4N_hydroxyl – 3N_(ether) – 5N_C=C + 3N_sulfone – 3N_acrylic ester – 5N_ (isolated saturated aliphatic hydrocarbon rings, i.e., cyclohexyl or cyclopentyl)**
**N_SP**	number of atoms in the **shortest path across the backbone** of a polymeric repeat unit, **N_SP ≤ N_BB**<br>(Counting the atoms which were in the shortest pathway from one monomer to the next monomer, need to pay attention to the polymer backbone structures)
**Nmv**	**Nmv = 2*N_ester + 3*N_ether**<br><br>**Ester**<br>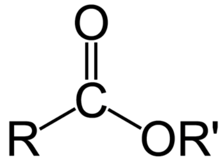<br>**Ether**<br>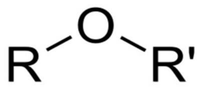
**$^0\chi$**	the **zeroth-order (atomic) connectivity indices** (the first atomic index)<br>It reflects the connectivity of non-hydrogen atoms attached to a given non-hydrogen atom. In the monomer structure, atoms are represented as vertices in a hydrogen-suppressed graph.
**$^0\chi^V$**	the **zeroth-order (atomic) connectivity indices** (the second atomic index)<br>It incorporates detailed valence electrons information for an atom, shows the electronic configuration of each non-hydrogen atom. In the monomer structure, atoms are represented as vertices in a hydrogen-suppressed graph.
**$^1\chi$**	the **first-order (bond) connectivity indices** (the first bond index)

	It reflects the bonding information of each bond that does not involve a hydrogen atom. In the monomer structure, bonds are represented as edges in a hydrogen-suppressed graph.
$^{1}\chi^{V}$	the **first-order (bond) connectivity indices** (the second bond index) It provides information about the electronic configuration of bonds that do not involve a hydrogen atom. In the monomer structure, bonds are represented as edges in a hydrogen-suppressed graph.
**BB_index1**	backbone index1 is a **steric hindrance parameter** that reflects the **flexibility** of the polymer backbone structure, similar to the stiffness of the backbone. It serves as an indicator of the extent to which the backbone bonds exhibit "hinge-like" behavior, influencing the overall flexibility and movement of the polymer chain.
**BB_index2**	backbone index2 is a **steric hindrance parameter** that differentiates between backbone atoms with the same (δ/δV) values but different δ values, reflecting **variations in the number of non-hydrogen neighbors** around each backbone atom.

S3. Feature Calculation Example

To illustrate the calculation of the four connectivity indices and two backbone indices, we use a polymer monomer structure as an example to walk through the detailed calculations and formulas.

Structure of the repeat unit of Poly (Acetaldehyde)

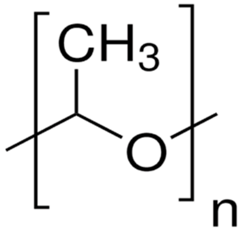

Find the corresponding (δ/δ^{V}) for every atom in the repeat unit and represent the vertices with (δ, δ^{V}).

Basic group	δ	δ^v	Basic group	δ	δ^v
-CH3	1	1	-NH2	1	3
-CH2-	2	2	-N-	2	5
=CH2	1	2	-N<	3	5
-CH<	3	3	-OH	1	5
>C<	4	4	=O	1	6
=CH-	2	3	-O-	2	6
=C<	3	4	-F	1	7
-NH-	2	4	-Cl	1	7/9

Then the hydrogen suppressed graph of the monomer is as below:

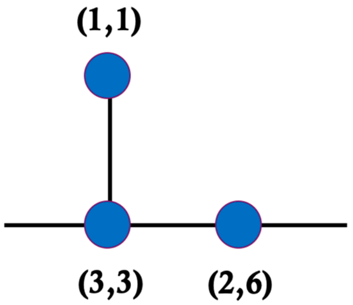

According to the formula of zeroth-order (atomic) connectivity indices:

$$^0\chi^V = \sum_{i \in N} \frac{1}{\sqrt{\delta_i^V}} \qquad ^0\chi = \sum_{i \in N} \frac{1}{\sqrt{\delta_i}}$$

$^0\chi = \frac{1}{\sqrt{1}} + \frac{1}{\sqrt{3}} + \frac{1}{\sqrt{2}} = 2.284$

$^0\chi^v = \frac{1}{\sqrt{1}} + \frac{1}{\sqrt{3}} + \frac{1}{\sqrt{6}} = 1.985$

According to the formula of first-order (atomic) connectivity indices:

$$^1\chi = \sum_{(i,i')\in\varepsilon} \frac{1}{\sqrt{\delta_i \delta_{i'}}} \qquad ^1\chi^V = \sum_{(i,i')\in\varepsilon} \frac{1}{\sqrt{\delta_i^V \delta_{i'}^V}}$$

$^1\chi = \frac{1}{\sqrt{1\times3}} + \frac{1}{\sqrt{3\times2}} = 0.985$

$^1\chi^v = \frac{1}{\sqrt{1\times3}} + \frac{1}{\sqrt{3\times6}} = 0.813$

According to the formula of backbone index1 and 2:

$$\text{BB\_index1} = \frac{\sum_{BBatoms}\left(\frac{\delta}{\delta V}\right)}{NBB}$$

$$\text{BB\_index2} = 3 - \frac{\sum_{BBatoms}(\delta)}{NBB}$$

NBB – the number of backbone atoms

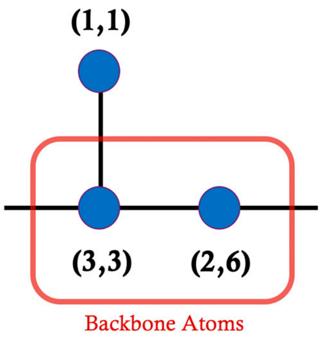

Calculate the backbone index 1 and 2 by looking at the backbone structure of the monomer:

$$\text{BB\_index1} = \frac{\frac{3}{3}+\frac{2}{6}}{2} = 0.67$$

$$\text{BB\_index2} = 3 - \frac{3+2}{2} = 0.5$$

S4. Feature Intercorrelations

The following heat maps show the heat map labeling the correlations between features, using only those features identified as most important, and then with all features considered. As discussed in the main text, some features are highly correlated with each other ($r > 0.80$), meaning they may carry redundant information:

- N_H strongly correlates with 0X ($r = 0.85$) and 1Xv ($r = 0.93$)
- 0X and 1Xv almost perfectly correlated ($r = 0.95$)
- Nmv negatively correlates with N_K ($r = -0.86$)

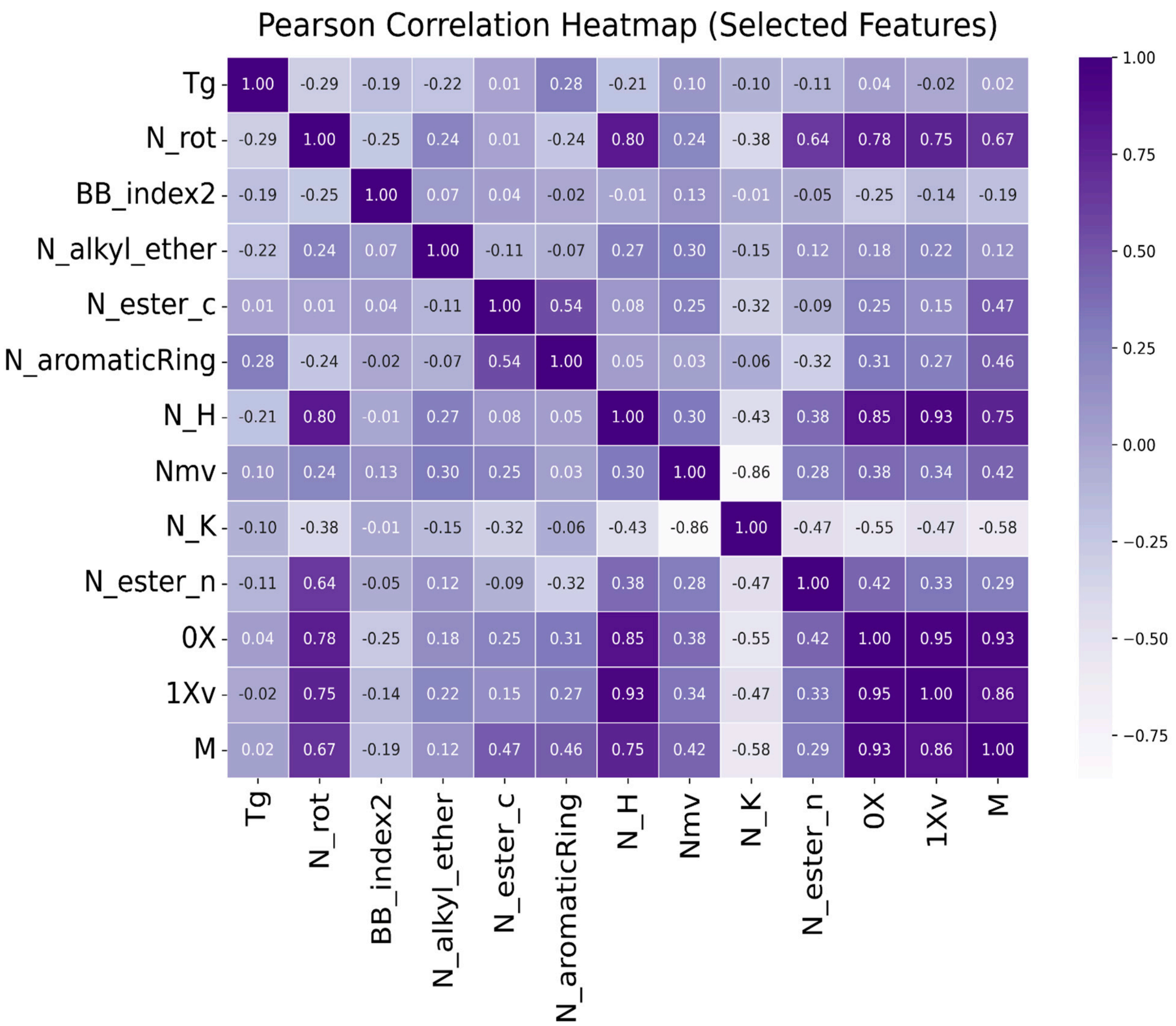
Pearson Correlation Heatmap (Selected Features)
Tg
N_rot
BB_index2
N_alkyl_ether
N_ester_c
N_aromaticRing
N_H
Nmv
N_K
N_ester_n
0X
1Xv
M
1.00 -0.29 -0.19 -0.22 0.01 0.28 -0.21 0.10 -0.10 -0.11 0.04 -0.02 0.02
-0.29 1.00 -0.25 0.24 0.01 -0.24 0.80 0.24 -0.38 0.64 0.78 0.75 0.67
-0.19 -0.25 1.00 0.07 0.04 -0.02 -0.01 0.13 -0.01 -0.05 -0.25 -0.14 -0.19
-0.22 0.24 0.07 1.00 -0.11 -0.07 0.27 0.30 -0.15 0.12 0.18 0.22 0.12
0.01 0.01 0.04 -0.11 1.00 0.54 0.08 0.25 -0.32 -0.09 0.25 0.15 0.47
0.28 -0.24 -0.02 -0.07 0.54 1.00 0.05 0.03 -0.06 -0.32 0.31 0.27 0.46
-0.21 0.80 -0.01 0.27 0.08 0.05 1.00 0.30 -0.43 0.38 0.85 0.93 0.75
0.10 0.24 0.13 0.30 0.25 0.03 0.30 1.00 -0.86 0.28 0.38 0.34 0.42
-0.10 -0.38 -0.01 -0.15 -0.32 -0.06 -0.43 -0.86 1.00 -0.47 -0.55 -0.47 -0.58
-0.11 0.64 -0.05 0.12 -0.09 -0.32 0.38 0.28 -0.47 1.00 0.42 0.33 0.29
0.04 0.78 -0.25 0.18 0.25 0.31 0.85 0.38 -0.55 0.42 1.00 0.95 0.93
-0.02 0.75 -0.14 0.22 0.15 0.27 0.93 0.34 -0.47 0.33 0.95 1.00 0.86
0.02 0.67 -0.19 0.12 0.47 0.46 0.75 0.42 -0.58 0.29 0.93 0.86 1.00
1.00
0.75
0.50
0.25
0.00
−0.25
−0.50
−0.75

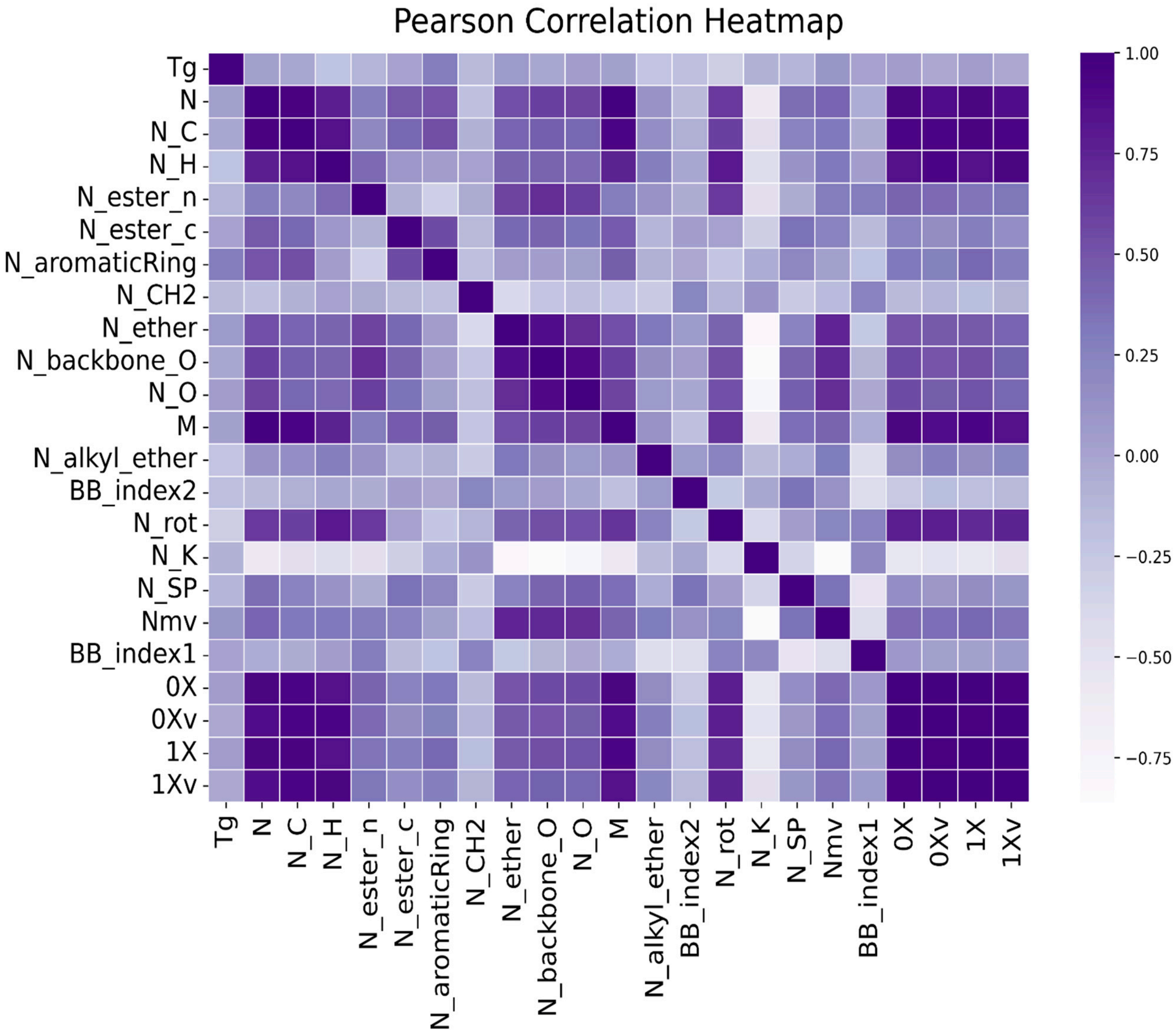
Pearson Correlation Heatmap
Tg
N
N_C
N_H
N_ester_n
N_ester_c
N_aromaticRing
N_CH2
N_ether
N_backbone_O
N_O
M
N_alkyl_ether
BB_index2
N_rot
N_K
N_SP
Nmv
BB_index1
0X
0Xv
1X
1Xv
1.00
0.75
0.50
0.25
0.00
−0.25
−0.50
−0.75
